# First detection of a *Vssc* allele V1016G conferring a high level of insecticide resistance in *Aedes albopictus* collected from Europe (Italy) and Asia (Vietnam), 2016: a new emerging threat to controlling arboviral diseases

**DOI:** 10.2807/1560-7917.ES.2019.24.5.1700847

**Published:** 2019-01-31

**Authors:** Shinji Kasai, Beniamino Caputo, Takashi Tsunoda, Tran Chi Cuong, Yoshihide Maekawa, Sai Gek Lam-Phua, Verena Pichler, Kentaro Itokawa, Katsunori Murota, Osamu Komagata, Chigusa Yoshida, Han-Hsuan Chung, Romeo Bellini, Yoshio Tsuda, Hwa-Jen Teng, José Luiz de Lima Filho, Luiz Carlos Alves, Lee Ching Ng, Noboru Minakawa, Nguyen Thi Yen, Tran Vu Phong, Kyoko Sawabe, Takashi Tomita

**Affiliations:** 1 Department of Medical Entomology, National Institute of Infectious Diseases, Tokyo, Japan; 2 Parasitology Unit, Department of Public Health and Infectious Diseases, Università di Roma ‘La Sapienza’, Rome, Italy; 3 Department of Vector Ecology and Environment, Nagasaki University, Nagasaki, Japan; 4 Medical Entomology and Zoology Department, National Institute of Hygiene and Epidemiology, Hanoi, Vietnam; 5 Environmental Health Institute, National Environment Agency, Singapore; 6 Department of Research Promotion, Japan Agency for Medical Research and Development, Tokyo, Japan; 7 Center for Diagnostics and Vaccine Development, Centers for Disease Control, Ministry of Health and Welfare, Taiwan; 8 Agriculture and Environment Centre “G. Nicoli”, Crevalcore, Italy; 9 Laboratório de Imunopatologia Keizo Asami, UFPE, Recife, Brazil; 10 Aggeu Magalhães Institute, FIOCRUZ, Recife, Brazil

**Keywords:** *Aedes albopictus*, pyrethroid resistance, voltage-sensitive sodium channel, *kdr*, dengue, V1016G

## Abstract

**Introduction:**

*Aedes albopictus* (Skuse) is an important vector of arboviral diseases, including dengue, chikungunya and Zika virus disease. Monitoring insecticide resistance and mechanisms by which the mosquito develops resistance is crucial to minimise disease transmission.

**Aim:**

To determine insecticide resistance status and mechanisms in *Ae. albopictus* from different geographical regions.

**Methods:**

We sampled 33 populations of *Ae. albopictus* from Asia, Europe and South America, and tested these for susceptibility to permethrin, a pyrethroid insecticide. In resistant populations, the target site for pyrethroids, a voltage-sensitive sodium channel (*Vssc*) was genotyped. Three resistant sub-strains, each harbouring a resistance allele homozygously, were established and susceptibilities to three different pyrethroids (with and without a cytochrome P450 inhibitor) were assayed.

**Results:**

Most populations of *Ae. albopictus* tested were highly susceptible to permethrin but a few from Italy and Vietnam (4/33), exhibited high-level resistance. Genotyping studies detected a knockdown resistance (*kdr*) allele V1016G in *Vssc* for the first time in *Ae. albopictus*. Two previously reported *kdr* alleles, F1534C and F1534S, were also detected. The bioassays indicated that the strain homozygous for the V1016G allele showed much greater levels of pyrethroid resistance than other strains harbouring F1534C or F1534S.

**Conclusion:**

The V1016G allele was detected in both**Asian and Italian* Ae. albopictus* populations, thus a spread of this allele beyond Italy in Europe cannot be ruled out. This study emphasises the necessity to frequently and regularly monitor the V1016G allele in *Ae. albopictus*, particularly where this mosquito species is the main vector of arboviruses.

## Introduction

The Asian tiger mosquito, *Aedes albopictus*, inhabits tropical and temperate regions and is an important vector of several arboviruses, including dengue, chikungunya and Zika viruses. To minimise the risk of human infection by such viruses, successful control of this mosquito and another important mosquito vector, *Aedes aegypti* (Linnaeus) is key. Mosquito control stongly relies on the use of insecticides, especially pyrethroids, that are known to have a rapid and high insecticidal activity, but low mammalian toxicity [1].

Insects develop resistance to insecticides by obtaining one or several of three resistance mechanisms: (i) reduced penetration of the insecticide through cuticles, (ii) increased level or activity of detoxification enzyme(s), such as cytochrome P450 monooxygenases (P450s) and carboxyl esterases, and/or (iii) reduced sensitivity of the target site [2].

The voltage-sensitive sodium channel (VSSC) is the target site for pyrethroids and dichlorodiphenyltrichloroethane (DDT). It is a membrane protein located on nerve axons, which consists of ca 2,000 amino acid residues. Some amino-acid substitutions in the VSSC are known to confer reduced sensitivity of the channel to pyrethroid insecticides. This type of resistance is generally called *knockdown resistance* or simply ‘*kdr*’ [3].

In general, *Ae. albopictus* has been observed to feed on a wide range of hosts and to mainly rest outdoors (exophily) while *Ae. aegypti* has a highly anthropophilic (preferring human blood) and endophilic (preferring human domestic environments) behaviours [4,5]. Thus, *Ae. aegypti* might be expected to be more frequently exposed to insecticides, possibly leading to a more rapid selection of resistant alleles in this species, and the development of higher levels of insecticide resistance compared to *Ae. albopictus*.

In line with this, several amino-acid substitutions responsible for* kdr* have been previously reported in* Ae. aegypti*, either solely or in combination with other substitutions: V1016G; F1534C; S989P + V1016G; V1016G + F1534C; S989P + V1016G + F1534C; V410L + F1534C [6-8].

Pyrethroids can be classified into two groups according to the absence (type I) or presence (type II) of the α-cyano group in their alcohol moiety [1]. Electrophysiological studies have shown that V1016G confers resistance to both type I and II pyrethroids [9,10]. This mutation also confers a higher level of resistance than F1534C to permethrin, a type I pyrethroid, while a VSSC with a F1534C substitution is sensitive to type II pyrethroids, such as deltamethrin and cypermethrin [9-11]. These electrophysiological observations, however, have never been verified by bioassays with strains carrying these alleles homozygously.

In contrast to* Ae. aegypti*, *Ae. albopictus* whose behaviour does not primarily depend upon a human living environment, thereby possibly reducing its insecticide exposure, has maintained relatively high susceptibility to pyrethroids [7]. Until 2011, when we reported the detection of F1534C allele in *Ae. albopictus* collected from Singapore, no *kdr* allele had been reported in this particular mosquito species [12]. Subsequently, the same allele was also detected in *Ae. albopictus* collected from Greece in 2016 [13] and Brazil in 2017 [14]. In 2016, another possible *kdr* allele, F1534S, was detected in *Ae. albopictus* collected from China and the United States [13,15]. Levels of pyrethroid resistance conferred by the F1534S and F1534C alleles, however, have never been studied. More recently, Pichler et al. reported first evidence of pyrethroid resistance in Italian *Ae. albopictus* [16] but the molecular mechanism of the resistance was not investigated.

In the current study, we tested pyrethroid susceptibilities for 33 *Ae. albopictus* populations collected from several geographical regions worldwide and the mechanisms conferring pyrethroid resistance were examined.

### Methods

#### Collection of mosquitoes

Larvae of *Ae. albopictus* were collected from fields of several countries including Brazil, Italy, Vietnam and Singapore. Mosquitoes were also sampled in Taiwan. Detailed information on populations used for bioassays are listed in Table 1.

**Table 1:** List of *Aedes albopictus* populations used for bioassay and genotyping studies, November 2015–March 2017 (n = 33 populations)

Population name in Figure 1^a^	Collection site	Code name	Latitude	Longitude	Collection date	Generation tested
Hanoi 1	Tu Liem, Hanoi, Vietnam	HNI-PK1	21.0	105.8	07-Jun-2016	G1
Hanoi 2	Tu Liem, Hanoi, Vietnam	HNI-PK6–9	21.0	105.8	07-Jun-2016	G1
Hanoi 3	Tu Liem, Hanoi, Vietnam	HNI-TL6	21.0	105.8	06-Jun-2016	G1
Hanoi 4	Tu Liem, Hanoi, Vietnam	HNI-TL5	21.0	105.8	07-Jun-2016	G1
Hanoi 5	Tu Liem, Hanoi, Vietnam	HNI-TL2, 4	21.0	105.8	07-Jun-2016	G1
Hanoi 6	Tu Liem, Hanoi, Vietnam	HNI-H3, 5	21.0	105.8	02-Feb-2016	G2
Hanoi 7	Tu Liem, Hanoi, Vietnam	HNI-TL1	21.0	105.8	07-Jun-2016	G3
Hanoi 8	Tu Liem, Hanoi, Vietnam	HNI-TL3	21.0	105.8	07-Jun-2016	G3
Hanoi 9	Bat Trang, Hanoi, Vietnam	HNI-BT2–7	21.0	105.9	02-Feb-2016	G1
Bavi 1	Ba Vi, Hanoi, Vietnam	BAV-3A, 3B	21.1	105.4	08-Jun-2016	G1
Bavi 2	Ba Vi, Hanoi, Vietnam	BAVI-2A, 2B	21.1	105.4	08-Jun-2016	G1
Bavi 3	Ba Vi, Hanoi, Vietnam	BAV-1A, 1B, 1C	21.1	105.4	08-Jun-2016	G1
Bavi 4	Ba Vi, Hanoi, Vietnam	BAV-4A	21.1	105.4	08-Jun-2016	G1
Bavi 5	Ba Vi, Hanoi, Vietnam	Bavi1–9	21.1	105.4	09-Jun-2016	G2
Ho Chi Minh 1	Ho Chi Minh city, Vietnam	HCM-Park1	10.8	106.7	12-Sep-2016	G2
Ho Chi Minh 2	Ho Chi Minh city, Vietnam	HCM-Park2, 3	10.8	106.7	16-Sep-2016	G1
Ho Chi Minh 3	Ho Chi Minh city, Vietnam	HCM-123–5	10.8	106.7	13-Sep-2016	G1
Ho Chi Minh 4	Ho Chi Minh city, Vietnam	HCM-123–1,2,3,4	10.8	106.7	13-Sep-2016	G1
Ho Chi Minh 5	Ho Chi Minh city, Vietnam	HCM-108–3,4,9	10.8	106.7	12-Sep-2016	G1
Cat Tien 1	Cat Tien National Park, Dong Nai, Vietnam	CT-12,14,17	11.4	107.4	15-Sep-2016	G2
Dak Lak 1	Yok Don National Park, Dak Lak, Vietnam	DL-P1, 2	12.7	107.7	22-Sep-2016	G2
Dak Lak 2	Yok Don National Park, Dak Lak, Vietnam	DL-F2–10	12.7	107.7	22-Sep-2016	G2
Dak Lak 3	Buon Ma Thuot, Dak Lak, Vietnam	DL-U1–1,2,3,4,5	12.7	108.0	19-Sep-2016	G3
Dak Lak 4	Yok Don National Park, Dak Lak, Vietnam	DL-F2–5	12.7	107.7	22-Sep-2016	G3
Hoa Kien	Hoa Kien, Phu Yen, Vietnam	Hoa Kien	13.1	109.2	Nov-2015	G4
Singapore	Singapore	SP16alb	1.4	103.9	2016	G2
Pingtung	Pingtung city, Taiwan	Pingtung	22.7	120.5	08-Mar-2016	G2
Tainan	Tainan city, Taiwan	Tainan	23.0	120.2	03-Mar-2016	G2
João Pessoa	João Pessoa, State of Paraíba, Brazil	João Pessoa	-7.1	-34.8	10-Mar-2016	G2
Maracanã	Maracanã, Rio de Janeiro, Brazil	Maracanã	-22.9	-43.2	13-Mar-2016	G2
Recife	Recife, State of Pernambuco, Brazil	RF	-8.1	-34.9	Mar-2017	G3
Emilia Romagna^b^	Lido di Spina, Emilia Romagna, Italy	Emilia Romagna	44.6	12.2	Sep to Oct, 2016	G0
Lazio	Verano, Lazio, Italy	Lazio	41.9	12.5	Sep to Oct, 2016	G1

^a ^The Emilia Romagna and Lazio populations are not represented in Figure 1.

^b ^Emilia Romagna population was previously used for the bioassay in Pichler et al. [16].

### Insecticides

Three pyrethroids were used. Two of these, permethrin (91.2%, Sumitomo Chemical Co., Ltd., Tokyo, Japan) and etofenprox (99.0%, Wako Pure Chemical Industries, Ltd., Osaka, Japan) were type I pyrethroids. The third, deltamethrin (99.4%, GL Sciences Inc., Tokyo, Japan), was a type II pyrethoid. Cytochrome P450 inhibitor, piperonyl butoxide (PBO, 98.0%) was obtained from Wako Pure Chemical Industries, Ltd., Osaka, Japan.

### Adult bioassay

Bioassays were conducted to assess the susceptibility of adult *Ae. albopictus* to pyrethroids (mated females). Topical applications were performed as described previously [17]. 

First, bioassays were carried out for a reference strain HKM. The HKM strain was collected originally from Tokyo, Japan in 2010. The 50% and 99% lethal dose (LD_50_ and LD_99_) values were calculated using Finney’s log-probit mortality regression analysis [18], implemented in R version 3.3.3 (www.r-project.org).

For testing susceptibilities of field-collected *Ae. albopictus* populations, treatments were performed with permethrin, 5.87 ng/female (i.e. LD_99_ for HKM) and 58.7 ng/female (i.e. LD_99_ x 10 for HKM) and mortalities were assessed 24 hours after treatments. For every given population, pools of 20 respective mosquitoes (4 to 6-days old) were treated in four replicates with each permethrin dose. All bioassays except for two Italian populations were conducted at the National Institute of Infectious Diseases, Japan.

To test the pyrethroid susceptibility of two Italian populations, the World Health Organization (WHO) standard filter paper method [19] containing 0.75% permethrin was employed as previously described [16] at Sapienza University of Rome. Approximately 25 adult females (3 to 5-days old) of the Lazio population were exposed in three replicates to the filter paper for 15 min and mortality was counted 24 hour after the treatment. For the Emilia Romagna population, approximately 25 females (3 to 5-days old) were used in three replicates and permethrin exposure was for 60 min as previously reported [16]. Permethrin exposure time was shortened for Lazio population to obtain sufficient number of survivors for testing phenotype–genotype association.

### Genotyping

After permethrin bioassays, genomic DNA was extracted from the legs of both dead and surviving mosquitoes using an alkaline method [20]. Partial genomic DNA was sequenced from domains II and III of the *Vssc* genes from seven populations exhibiting relatively low permethrin susceptibilities: Hanoi 1; Hanoi 2; Ho Chi Minh 1; Singapore; Pingtung; Emilia Romagna; and Lazio. Three possible *kdr* mutation sites reported from *Aedes*: S989P; V1016G/I; F1534C/S were genotyped by PCR and direct sequencing. Two other mutation sites, I1011M/V [8] and I1532T [13], reported from pyrethroid resistant *Ae. aegypti* were also investigated. In this paper, we numbered the amino acid position according to the sequence of the most abundant splice variant of the house fly *Vssc* (GenBank accession number: AAB47604). Detailed procedures for genotyping have been described previously [17], with the exception of our use of the primer AaSCF9 instead of AaSCF3 for sequencing domain II (Supplement S1). For genotyping the M944V allele in the alternative exon D [21], females of SusSP16 were exposed to 0.033 ng of deltamethrin and PCR was performed for 31 surviving and eight dead mosquitoes. The albSCF10 and albSCR12 primers were used for PCR and then the PCR products were directly sequenced with albSCF11. The Fisher’s exact test was used to assess the significance of the associations between *kdr* genotype and permethrin susceptibility.

### Establishment of homozygous strains

To establish resistant strains harbouring single *kdr* alleles homozygously, the Hanoi 2 (PK69) and Singapore (SP16) populations were genotyped and each population selectively crossed. Pupae were isolated individually in 2.0 mL Eppendorf tubes. After these had developed into adults, one hind leg was removed and genomic DNA was isolated. Each mosquito was placed into another 2.0-mL Eppendorf tube with a piece of water-saturated cotton until genotyping analysis was complete. Genotyping was carried out for V1016G and F1534C/S alleles as described above. 

For the Hanoi 2 population, 126 virgin females and 71 males were genotyped. Fifteen females and eight males were V1016G allele homozygotes, and we established a sub-strain designated as PK69G from their progenies. Thirty-three females and 22 males, on the contrary, had no *kdr* alleles. Their progenies were designated as SusPK69.

The Hanoi 2 population also contained the F1534S allele at low frequency. To obtain homozygous individuals, a two-step procedure was employed. First, F1534S/F1534 heterozygotes (11 females and five males) were crossed. In the next generation, mosquitoes with F1534S allele homozygotes (five females and nine males of 128 and 112, respectively) were crossed for establishing PK69S. 

For the Singapore population, 140 virgin females and 96 males were genotyped. Eighty-eight females and 59 males which were F1534C homozygotes were crossed for establishing SP16C sub-strain. Twelve females and eight males with no *kdr* alleles were also crossed for establishing SusSP16.

Bioassays were carried out to obtain LD_50_s for isolated strains against permethrin, etofenprox, and deltamethrin as described above. To estimate the contribution of insecticide resistance by P450s, 2 μg of PBO, which was a sub-lethal dose for HKM, was applied to mosquitoes 2 hours before exposing with pyrethroids.

### Species identification using mitochondria cytochrome c oxidase subunit I gene

Partial mitochondrial cytochrome c oxidase subunit I (*COI*) genes were sequenced to confirm the mosquito species of six sub-strains (4 individuals from each strain) established in this study (HKM, SusPK69, PK69G, PK69S, SusSP16 and SP16C) as described previously [22].

### Next generation sequencing of the *Vssc* cDNA

Full-length *Vssc* cDNAs from eight (SusSP16) or four (other strains) virgin females were individually sequenced. RNA was isolated using Isogen II (Nippon Gene Co., Ltd., Tokyo, Japan) and cDNA was synthesised using total RNA, a poly-T primer and an AccuScript High Fidelity 1st Strand cDNA Synthesis Kit (Agilent Technologies, California (CA), United States (US)). Full-length cDNAs of *Vssc* were amplified by PCR in two overlapping pieces (5´ and 3´ ends). For the 5´ end, albSCF1 and albSCR9 primers were used, and for the 3´ end, albSCF6 and NavAlR22 primers were used (Supplement S1). PCR was performed using PrimeSTAR GXL DNA Polymerase (Takara Bio Inc., Shiga, Japan) under the following conditions: an initial denaturation at 95 °C for 2 min; followed by 38 cycles at 98 °C for 10 s; 60 °C for 15 s; 68 °C for 3 min; with a final extension at 68 °C for 10 min. The 5’- and 3’-cDNA fragments of each mosquito was pooled, sheared, and ligated with same indexed adaptor. After heat inactivating the ligase, all samples of different mosquitoes were pooled, purified with 1XSPRIselect (Beckman Coulter, Brea, CA, US) and fragments of ca 500 to 800 bp DNA were sequenced using the Illumina MiniSeq system (Illumina) in 150 bp paired-end mode using Mini Seq Mid Output kit (Illumina). Illumina data were analysed using the CLC Genomics Workbench ver. 10.0.1 (Qiagen, Venlo, the Netherlands).

## Results

### Susceptibility to permethrin of field-collected populations

**Figure 1:**
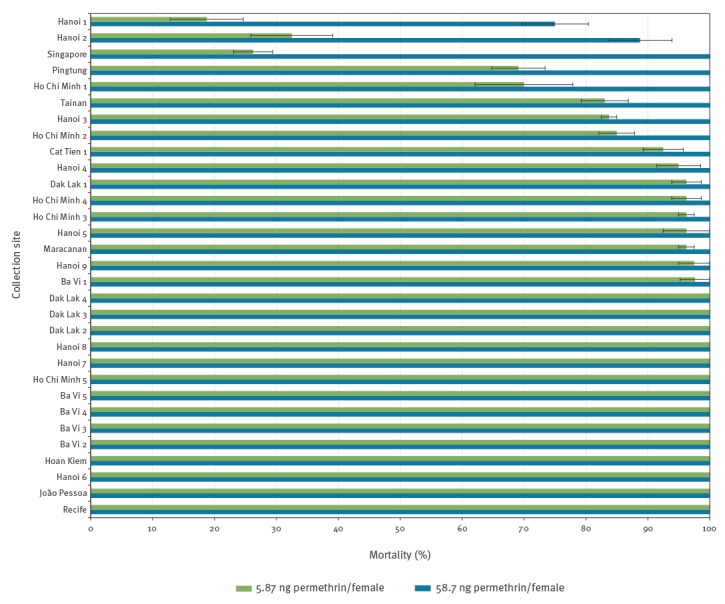
Mortalities of adult *Aedes albopictus* after exposures to permethrin, September 2015–June 2017 (n = 31 populations)^a^

At permethrin doses of 5.87 ng/female (i.e. LD_99_ of HKM), 14 of 31 populations exhibited 100% mortality, nine populations exhibited between 90% and 100% mortality and five populations (Hanoi 1, Hanoi 2, Ho Chi Minh 1, Singapore and Pingtung) exhibited < 80% mortality (Figure 1). When doses were increased to 58.7 ng/female, all populations, except two from Vietnam, exhibited 100% mortality. Hanoi 1 and Hanoi 2 populations showed mortalities of 75.0% and 88.8%, respectively, under the higher dose.

From each mosquito population, groups of females were topically exposed to two doses of permethrin, i.e. 5.87 ng or 58.7 ng, which were respectively equivalent to the LD_99_ and LD_99_x10 doses for the reference strain HKM. Twenty females were simultaneously treated in each assay, whereby the subsequent proportion of dead mosquitoes was calculated. Data, which are presented, are the average of four replicates. Error bars indicate standard errors for the mortalities of each population.

^a^ Data from the Emilia Romagna and Lazio populations are not shown in the Figure as these were obtained by a different method. The bioassay from Pichler et al. [16] was used for the Emilia Romagna population.

Lazio population exhibited 77.1% ± 7.5% mortality by WHO filter paper method. As was previously reported, Emilia Romagna population exhibited 81.3% ± 15% mortality [16]. According to WHO guidelines, populations were considered ‘resistant’ if mortality was < 90% upon pyrethroid exposure for 60 min [16].

### Genotyping of *Vssc*

Partial genomic DNA fragments of *Vssc* were sequenced from the seven populations that showed relatively low susceptibilities to permethrin. Genotypes and frequencies of *Vssc* alleles are shown in Table 2. Genotyping results are deposited to VectorBase.org (Project ID: VBP0000370). The V1016G allele was detected in four populations: Emilia Romagna and Lazio (both Italy); Hanoi 1 and Hanoi 2 (both Vietnam). This was the first detection of the V1016G allele from *Ae. albopictus*. In all populations, frequencies of the V1016G allele were higher in surviving animals than in dead ones and the genotype G1016 showed significant association (p < 0.01) to permethrin resistance in all Lazio, Hanoi 1, and Hanoi 2 populations (Table 2). All survivors of Hanoi 1 population exposed to permethrin of LD_99_X10 were homozygous for the V1016G allele.

**Table 2:** Genotype of voltage-sensitive sodium channel in seven populations of *Aedes albopictus*, February 2016–November 2017 (n = 293 mosquitoes)

Genotype (1016–1532–1534^a^)	Emilia Romagna		Lazio		Hanoi 1		Hanoi 2		Ho Chi Minh 1		Singapore		Pingtung	
	Dead^b^	Alive^b^	Dead^c^	Alive^c^	Dead^d^	Alive^d^	Dead^e^	Alive^e^	Dead^e^	Alive^e^	Dead^e^	Alive^e^	Dead^e^	Alive^e^
V/V–I/I–F/F	2	0	0	0	6	0	9	4	17	8	3	0	24	24
V/V–I/T–F/F	2	1	2	0	0	0	0	0	0	0	0	0	0	0
V/V–T/T–F/F	3	1	0	0	0	0	0	0	0	0	0	0	0	0
V/G–I/I–F/F	1	2	1	2	19	0	13	33	0	0	0	0	0	0
V/G–I/T–F/F	2	6	0	2	0	0	0	0	0	0	0	0	0	0
G/G–I/I–F/F	0	2	0	6	2	20	0	12	0	0	0	0	0	0
G/G–T/T–F/F	0	0	0	1	0	0	0	0	0	0	0	0	0	0
V/V–I/I–F/C	0	0	0	0	0	0	0	0	7	7	11	9	0	0
V/V–I/I–C/C	0	0	0	0	0	0	0	0	0	3	6	15	0	0
V/V–I/I–F/S	0	0	0	0	0	0	1	1	0	0	0	0	0	0
V/V–I/I–S/S	0	0	0	0	0	0	0	1	0	0	0	0	0	0
V/G–I/I–F/S	0	0	0	0	0	0	0	2	0	0	0	0	0	0
n^f^	10	12	3	11	27	20	23	53	24	18	20	24	24	24
*P* value^g^ for G1016	0.0245		0.00658		1.84 x 10 ^− 10^		0.00250		NA		NA		NA	
*P* value^g^ for T1532	0.543		0.459		NA		NA		NA		NA		NA	
*P* value^g^ for C1534	NA		NA		NA		NA		0.0369		0.0195		NA	
*P* value^g^ for S1534	NA		NA		NA		0.668		NA		NA		NA	

NA: not applicable; WHO: World Health Organization.

^a ^Each mosquito has two alleles (maternal and paternal) for each position of the amino acid.

^b ^WHO test (0.75% permethrin), exposed for 60 min.

^c ^WHO test (0.75% permethrin), exposed for 15 min.

^d ^Topical application (58.7 ng of permethrin; LD_99_X10 for HKM).

^e ^Topical application (5.87 ng of permethrin; LD_99_ for HKM).

^f ^n: Number of females tested.

^g ^Fisher’s exact test (number of alleles (wild type or mutated) vs permethrin susceptibility (dead or surviving mosquito)).

The F1534C allele was detected in the Ho Chi Minh 1 and Singapore populations. The genotype C1534 showed weak associations to permethrin resistance in Ho Chi Minh 1 and Singapore populations (p = 0.0369 and 0.0195, respectively) (Table 2). The F1534S allele was detected in the Hanoi 2 population in the current study as well. There is so far no evidence of a double mutation V1016G + F1534S. Other possible *kdr* alleles S989P or I1011M were not detected in this study. We also did not detect the L1014F allele, which is the most common *kdr* mutation found in many insects but has never been detected from *Aedes* mosquitoes so far [8].

### Establishment of homozygous strains

Five sub-strains were established in this study. The PK69G, PK69S and SusPK69 sub-strains were isolated from the Hanoi 2 population (originally designated as PK69), while the SP16C and SusSP16 sub-strains were isolated from the Singapore population (originally designated as SP16). The *COI* analysis revealed that all sequences showed the highest homology scores to *Ae. albopictus* genes using Nt Basic Local Alignment Search Tool (BLAST) search (data not shown).

Genotyping of 24 mosquitoes from each sub-strain confirmed that PK69G, PK69S and SP16C possess the V1016G, F1534S, or F1534C alleles, respectively and homozygously.

### Contribution of each *kdr* allele to pyrethroid resistance

**Figure 2:**
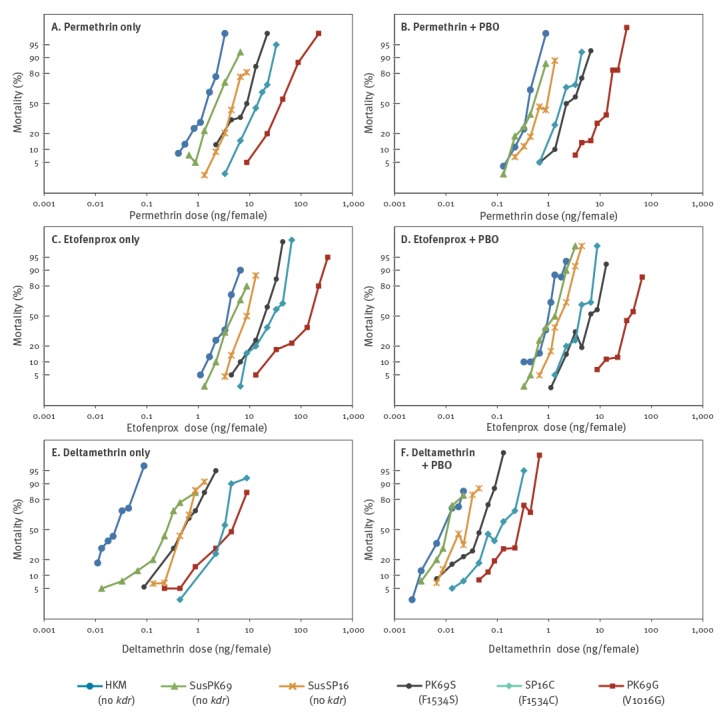
Log dosage-probit mortality lines of six *Aedes albopictus* strains topically exposed to: (A, B) permethrin, (C, D) etofenprox and (E, F) deltamethrin, September 2015–October 2017 (n = 12,145 mosquitoes)

We carried out bioassays with three pyrethroids (i.e. deltamethrin, etofenprox and permethrin) against HKM and the five established sub-strains (PK69G, PK69S, SusPK69, SP16C and SusSP16). Dose-probit mortality lines are shown in Figure 2. Slopes, LD_50_s, resistance ratios and the synergistic ratios of PBO are shown in Table 3. The LD_50_ of HKM to permethrin was 1.26 ng/female which was lower than the LD_50_ of SMK (1.48 ng/female), an insecticide susceptible strain of *Ae. aegypti* (Table 3) [17].

PBO: piperonyl butoxide.

PBO (2 μg) was applied 2 hours before the pyrethroid treatment to inhibit detoxification by the detoxification enzyme cytochrome P450s (B, D, F). SusPK69, PK69G and PK69S were isolated from Hanoi 2 population, and SusSP16 and SP16C were isolated from Singapore population. SMK is an insecticide-susceptible strain of *Aedes aegypti* [17].

**Table 3:** Toxicity of three pyrethroids against six strains of *Aedes* *albopictus* having different voltage-sensitive sodium channel alleles (PK69G, PK69S, SP16C) or no mutation (HKM, SusPK69, SusSP16), September 2015–October 2017 (n = 12,145 mosquitoes)

Strain	n	Slope ± SE	LD_50_ (95%CI)	RR1^a^	RR2^b^	RR3^c^	SR^d^	Ratio of SR (vs HKM)
**Permethrin only**								
HKM	341	3.5 ± 0.30	1.26 (1.13–1.41)	1.0	NA	NA	NA	NA
SusPK69	200	3.2 ± 0.35	2.26 (1.91–2.66)	1.8	1	NA	NA	NA
PK69G	240	2.8 ± 0.33	39.0 (32.5–46.9)	31	17	NA	NA	NA
PK69S	240	2.8 ± 0.35	7.25 (6.25–8.41)	5.8	3.2	NA	NA	NA
SP16C	200	3.1 ± 0.41	14.4 (12.4–16.8)	11	NA	2.9	NA	NA
SusSP16	303	3.8 ± 0.39	4.92 (4.44–5.45)	3.9	NA	1	NA	NA
SMK^e^	354	7.0 ± 0.65	1.48 (1.40–1.56)	1.2	NA	NA	NA	NA
**Permethrin + PBO**								
HKM	340	4.4 ± 0.45	0.406 (0.366–0.449)	1.0	NA	NA	3.1	1.0
SusPK69	487	3.5 ± 0.28	0.461 (0.421–0.506)	1.1	1	NA	4.9	1.6
PK69G	460	3.3 ± 0.27	11.7 (10.6–12.9)	29	25	NA	2.8	0.90
PK69S	287	3.2 ± 0.36	2.64 (2.34–2.98)	6.5	5.7	NA	2.7	0.87
SP16C	200	3.7 ± 0.40	1.89 (1.65–2.15)	4.7	NA	2.6	7.6	2.5
SusSP16	311	3.5 ± 0.36	0.735 (0.661–0.818)	1.8	NA	1	6.7	2.1
SMK^e^	300	5.8 ± 0.59	0.890 (0.835–0.955)	2.2	NA	NA	1.7	0.55
**Etofenprox only**								
HKM	341	4.2 ± 0.34	3.27 (2.98–3.58)	1.0	NA	NA	NA	NA
SusPK69	200	3.4 ± 0.42	4.85 (4.19–5.60)	1.5	1	NA	NA	NA
PK69G	220	2.1 ± 0.27	117 (93.6–145)	36	24	NA	NA	NA
PK69S	213	3.7 ± 0.39	16.8 (14.6–19.4)	5.1	3.5	NA	NA	NA
SP16C	310	3.0 ± 0.28	25.8 (22.7–29.4)	7.9	NA	3.3	NA	NA
SusSP16	216	4.6 ± 0.49	7.91 (7.12–8.78)	2.4	NA	1	NA	NA
**Etofenprox + PBO**								
HKM	400	4.0 ± 0.34	0.922 (0.843–1.01)	1.0	NA	NA	3.5	1.0
SusPK69	280	3.8 ± 0.37	1.11 (0.99–1.24)	1.2	1.0	NA	4.4	1.3
PK69G	508	2.9 ± 0.23	35.8 (31.6–40.5)	39	32	NA	3.3	0.94
PK69S	378	2.6 ± 0.26	5.94 (5.16–6.84)	6.4	5.4	NA	2.8	0.80
SP16C	240	3.6 ± 0.42	4.10 (3.65–4.62)	4.4	NA	2.4	6.3	1.8
SusSP16	253	4.5 ± 0.45	1.68 (1.51–1.86)	1.8	NA	1	4.7	1.3
**Deltamethrin only**								
HKM	320	2.8 ± 0.29	0.0230 (0.0202–0.0263)	1.0	NA	NA	NA	NA
SusPK69	460	1.9 ± 0.19	0.249 (0.210–0.296)	11	1	NA	NA	NA
PK69G	240	1.9 ± 0.19	3.22 (2.57–4.05)	140	13	NA	NA	NA
PK69S	309	2.3 ± 0.19	0.470 (0.389–0.569)	20	1.9	NA	NA	NA
SP16C	220	3.4 ± 0.40	2.80 (2.40–3.25)	122	NA	5.7	NA	NA
SusSP16	221	3.3 ± 0.35	0.492 (0.424–0.570)	21	NA	1	NA	NA
**Deltamethrin + PBO**								
HKM	400	2.7 ± 0.23	0.00917 (0.00808–0.0104)	1.0	NA	NA	2.5	1.0
SusPK69	280	4.1 ± 0.39	0.00929 (0.00833–0.0103)	1.0	1	NA	27	11
PK69G	593	2.4 ± 0.18	0.229 (0.203–0.258)	25	25	NA	14	5.6
PK69S	463	2.4 ± 0.19	0.0373 (0.0327–0.0425)	4.1	4.0	NA	13	5.2
SP16C	400	2.1 ± 0.21	0.0969 (0.0833–0.113)	11	NA	4.3	29	12
SusSP16	417	3.4 ± 0.36	0.0226 (0.0207–0.0248)	2.5	NA	1	22	8.8

CI: confidence interval; n: number of mosquitoes tested; NA: not applicable; PBO: piperonyl butoxide; RR: resistance ratio; SE: standard error; SR: synergistic ratio.

^a ^RR1 = LD_50_ (each strain) / LD_50_ (HKM).

^b ^RR2 = LD_50_ (each strain) / LD_50_ (SusPK69).

^c ^RR3 = LD_50_ (SP16C) / LD_50_ (SusSP16).

^d ^SR = LD_50_ (without PBO) / LD_50_ (with PBO).

^e ^SMK is an insecticide-susceptible strain of *Aedes aegypti *[17].

When we assayed only for pyrethroid resistance, PK69G exhibited the highest resistance level of all of the six strains to all three pyrethroids. PK69G and SP16C exhibited particularly high levels of resistance to deltamethrin; resistance ratios were 140- and 122-fold, respectively, compared with the HKM strain. SusPK69 and SusSP16, neither of which possesses *kdr* alleles, also showed resistance ratios to deltamethrin of 11- and 21-fold, respectively, compared with the HKM strain. 

Synergistic ratios of PBO on deltamethrin toxicity were 11- and 8.8-fold higher in the SusPK69 and SusSP16 strains, respectively than HKM. 

Resistance ratios (pyrethroids + PBO) in PK69G and PK69S were 25- to 32-fold and 4.0- to 5.7-fold, respectively compared with the SusPK69 strain. In SP16C strain, resistance ratios (pyrethroids + PBO) were 2.4- to 4.3-fold compared with the SusSP16 strain (Table 3). Therefore, the strain harbouring V1016G allele (PK69G) showed several fold higher resistance levels than the strains harbouring F1534S allele (PK69S) or F1534C allele (SP16C).

### Other amino acid changes

Full length *Vssc* cDNAs were individually sequenced using Illumina MiniSeq NGS (Table 4). Results were submitted to Sequence Read Archive, SRA (BioProject ID: PRJDB6889). All 12 mosquitoes of PK69G, PK69S and SP16C possessed the V1016G, F1534S, or F1534C alleles, respectively and homozygously. In contrast, HKM, SusPK69 and SusSP16 did not have any alleles at these three amino acid loci. On the other hand, M686I, E832D and M944V (in the alternative exon D [21]) alleles were detected from HKM and SusSP16 strains. Since M944V was detected at a relatively high frequency in the SusSP16 strain, we genotyped this allele for both dead and surviving mosquitoes after deltamethrin (+ PBO) selection. The frequency of M944V allele in dead and surviving insects was 33/62 and 10/16, respectively, and there was no statistically significant difference observed between these two populations (p = 0.581).

**Table 4:** Mutations found in full length voltage-sensitive sodium channel protein in six strains of *Aedes albopictus*, June 2017–October 2017 (n = 28 mosquitoes)

Individual number	Mutations								
	K427R	M686I	E832D	M944V (Exon D^a^)	V1016G	F1534C/S	T2002P	A2023T	G2054GGG
**HKM (susceptible strain)**									
#1	K/R	M/M	E/E	M/M	V/V	F/F	T/T	A/A	G/G
#2	K/R	M/M	E/E	M/M	V/V	F/F	T/T	A/A	G/GGG
#3	K/R	M/M	E/E	M/M	V/V	F/F	T/T	A/A	G/G
#4	K/R	M/I	E/E	M/M	V/V	F/F	T/T	A/A	G/GGG
**SusPK69 (PK69 having no *kdr* mutation)**									
#1	K/R	M/M	E/E	M/M	V/V	F/F	T/T	A/A	G/G
#2	K/R	M/M	E/E	M/M	V/V	F/F	T/P	A/A	G/G
#3	K/R	M/M	E/E	M/M	V/V	F/F	T/T	A/A	G/G
#4	K/R	M/M	E/E	M/M	V/V	F/F	T/T	A/A	G/G
**PK69G (PK69 having V1016G mutation)**									
#1	K/R	M/M	E/E	M/M	G/G	F/F	T/T	A/A	G/GGG
#2	K/R	M/M	E/E	M/M	G/G	F/F	T/T	A/A	G/G
#3	K/R	M/M	E/E	M/M	G/G	F/F	T/T	A/A	G/GGG
#4	K/R	M/M	E/E	M/M	G/G	F/F	T/T	A/A	G/G
**PK69S (PK69 having F1534S mutation)**									
#1	K/R	M/M	E/E	M/M	V/V	S/S	T/T	A/T	G/G
#2	K/R	M/M	E/E	M/M	V/V	S/S	T/T	A/T	G/G
#3	K/R	M/M	E/E	M/M	V/V	S/S	T/T	A/T	G/G
#4	K/R	M/M	E/E	M/M	V/V	S/S	T/T	A/T	G/G
**SusSP16 (SP16 having no *kdr* mutation)**									
#1	K/R	M/M	E/E	V/V	V/V	F/F	T/T	A/A	G/G
#2	K/R	M/M	E/E	M/V	V/V	F/F	T/T	A/A	G/G
#3	K/R	M/M	E/D	M/V	V/V	F/F	T/T	A/A	G/G
#4	K/R	M/M	E/D	M/M	V/V	F/F	T/T	A/A	G/G
#5	K/R	M/M	E/E	V/V	V/V	F/F	T/T	A/A	G/G
#6	K/R	M/M	E/E	M/V	V/V	F/F	T/T	A/A	G/G
#7	K/R	M/M	E/E	M/V	V/V	F/F	T/T	A/A	G/G
#8	K/R	M/M	E/E	V/V	V/V	F/F	T/T	A/A	G/G
**SP16C (SP16 having F1534C mutation)**									
#1	K/R	M/M	E/E	M/V	V/V	C/C	T/T	A/T	G/G
#2	K/R	M/M	E/E	M/M	V/V	C/C	T/T	A/T	G/G
#3	K/R	M/M	E/E	M/M	V/V	C/C	T/T	A/T	G/G
#4	K/R	M/M	E/E	M/M	V/V	C/C	T/T	A/T	G/G

^a ^Alternatively spliced exon D as designated in the previous study [21].

In addition, the T2002P and A2023T alleles and an insertion of double glycine, were detected from the HKM, SusPK69, PK69S, SP16C and PK69G strains, all heterozygously. The E832D allele was detected in two of eight mosquitoes heterozygously in SusSP16. The K427R allele was detected heterozygously from all 28 mosquito samples tested.

## Discussion

Only two *kdr* alleles, F1534C and F1534S, have been reported from *Ae. albopictus* to date. In this study, a stronger *kdr* allele, V1016G, was detected in four *Ae. albopictus* populations collected from Italy and Vietnam, and its frequency was highly correlated with pyrethroid resistance. Thus, the present data are the first evidence of the V1016G allele from this mosquito species. It is speculated that V1016G allele was associated with the recent detection of pyrethroid resistant *Ae. albopictus* from Italy [16]. Previous electrophysiological studies have indicated that the V1016G allele in VSSC resulted in lower sensitivities to pyrethroids than F1534C [9-11], even though this was never evidenced in bioassays with living, homozygous strains. In this study, we established three new resistant sub-strains, each homozygously harbouring the V1016G, F1534C and F1534S alleles. Bioassays using PBO revealed that the V1016G allele was associated with greater pyrethroid resistance than the F1534C and the F1534S, which supports previous electrophysiological studies [9-11].

We also detected the I1532T allele from the Emilia Romagna and Lazio populations, as previously reported [13]. There was, however, no association between this allele and permethrin susceptibility (p > 0.4, Table 2). In addition, T2002P and A2023T alleles were newly found. These mutations, however, are unlikely to confer pyrethroid resistance since they are located near the C-terminus of the VSSC, where a polymorphic region is common among insect species. Curiously, the K427R allele was detected from all 28 mosquito samples tested by next generation sequencing. At this point we cannot provide any concrete explanation for this observation. One possible reason is RNA editing, as has been reported in previous studies on insect sodium channels [23]. The SusPK69 and SusSP16 strains, both of which have no *kdr* allele, showed 11- and 8.8-folds higher synergistic ratio on deltamethrin toxicity than that of HKM strain suggesting that P450 was certainly involved in the resistance in both the Hanoi 2 and Singapore populations. The Pingtung population showed a relatively lower susceptibility to permethrin, however, we did not detect any *kdr* allele within the *Vssc* regions sequenced, indicating the possibility of unknown *kdr* mutation or another resistance mechanism.

In this study, the *Vssc* harbouring the V1016G allele was not detected from *Ae. albopictus* collected outside of Hanoi City in Vietnam. As shown by our bioassay results, most of the populations collected in Vietnam, other than Hanoi 1 and Hanoi 2, exhibited high susceptibilities to permethrin, with the exception of one population from Ho Chi Minh City that included only a weak *kdr* F1534C at a relatively low frequency. Therefore, there is no evidence that the V1016G allele has spread widely in Asia. If that is the case, this raises several questions: (i) Why was this rare allele detected in mosquitoes collected from Italy?; and (ii) when and how was such a mosquito introduced into this country? It has been speculated that the origin of *Ae. albopictus* is south-east Asia, and that its distribution area spreads across Asia and into Europe via various imported items, such as used tyres and plant pods [24,25]. After the first chikungunya endemic in 2007 [26], pyrethroids have been frequently used for controlling adult *Aedes* mosquitoes in Italy [27]. It is possible that a very small number of mosquitoes with the V1016G allele were included in the initial population(s) of Italy and were gradually selected by pyrethroids, such that the allele frequency finally reached a level to which it could confer resistance. On the other hand, we cannot exclude the possibility that V1016G mutation occurred independently in Italy and Vietnam. In any case, the fact that the V1016G allele was detected in both northern (Emilia Romagna) and central (Lazio) Italy suggests, at least, that the introduction or appearance of this allele into the country occurred some time ago. It is further possible that *Ae. albopictus* mosquitoes harbouring the V1016G allele have spread already to other European countries; we therefore, emphasise the necessity to monitor this *kdr* allele in *Ae. albopictus* populations throughout Europe and other regions inhabited by *Ae. albopictus*. Furthermore, it is known that additional amino acid substitution can synergistically enhance effect of a *kdr* mutation [28,29]. An electrophysiological study has revealed that triple mutations of S989P, V1016G and F1534C in VSSC confer much greater levels of pyrethroid insensitivity than each single mutation [9] and *Ae. aegypti* mosquitoes harbouring these alleles actually have been found in several Asian countries [7]. The possibility exists that double or triple mutations including V1016G and another (other) *kdr* mutation(s) could also emerge in *Ae. albopictus.*

Recently, in chikungunya virus from the Indian Ocean, an alanine to valine mutation was found at position 226 in the E1 envelope glycoprotein, which caused a more than 100-fold increase in the vector competence of *Ae. albopictus* for this virus variant [30]. Autochthonous dengue outbreaks in Croatia (2010), Japan (2014), China (2014) and France (2015) were vectored by *Ae. albopictus* [31-34] and demonstrated the importance of this mosquito species for arbovirus transmission.

Development of insecticide resistance in *Ae. albopictus* had not been noted until recently, though the problem in *Ae. aegypti* has been well established and has become a priority concern in many countries [6,7]. However, since a strong *kdr* allele V1016G has been detected in *Ae. albopictus*, resistance in this species should also be considered. With the increased use of pyrethroids, the high level of insecticide resistance could potentially become widespread. Actions to prevent or delay this therefore need to be taken.

Multiple electrophysiological studies have suggested that the F1534C allele confers resistance to type I pyrethroids, but not to type II pyrethroids [9-11]. In this study, the SP16C strain harbouring the F1534C allele exhibited resistance not only to the type I pyrethroid permethrin (+ PBO) but also 4.3-fold of resistance to the type II pyrethroid deltamethrin (+ PBO), compared with the SusSP16 strain. It is suspected that this was simply due to the F1534C allele, because SP16C and SusSP16 strains were isolated from the same Singapore population and are supposed to share a similar genetic background. So, this result may suggest that F1534C confers resistance to type II pyrethroids as well. At this point, on the other hand, we cannot completely exclude the possibility that the effect was also due to another mutation hitchhiking near *Vssc* gene. A further study is needed to confirm this. Use of a congenic strain, which is established by backcrossing a *kdr*-type strain with a susceptible strain for several generations, may help to answer this question [35].

In conclusion, we report here for the first time detection of a *Vssc* allele, V1016G, in the mosquito *Ae. albopictus*. This allele was detected not only in *Ae. albopictus* collected from Asia (Vietnam), but also from Europe (Italy). In our study the V1016G allele conferred a higher level of pyrethroid resistance than the previously known alleles, F1534C and F1534S. As this could be relevant for the control of *Ae. albopictus*, we highlight the importance of monitoring this allele, especially in temperate regions where this insect species is the major vector of arboviral pathogens.

## Supplementary Data

27000 Supplement S1
